# Highly Sensitive In Vitro Methods for Detection of Residual Undifferentiated Cells in Retinal Pigment Epithelial Cells Derived from Human iPS Cells

**DOI:** 10.1371/journal.pone.0037342

**Published:** 2012-05-17

**Authors:** Takuya Kuroda, Satoshi Yasuda, Shinji Kusakawa, Naoya Hirata, Yasunari Kanda, Kazuhiro Suzuki, Masayo Takahashi, Shin-Ichi Nishikawa, Shin Kawamata, Yoji Sato

**Affiliations:** 1 Division of Cellular and Gene Therapy Products, National Institute of Health Sciences, Tokyo, Japan; 2 Foundation for Biomedical Research and Innovation, Kobe, Japan; 3 Division of Pharmacology, National Institute of Health Sciences, Tokyo, Japan; 4 Laboratory for Retinal Regeneration, RIKEN Center for Developmental Biology, Kobe, Japan; 5 Laboratory of Stem Cell Research, RIKEN Center for Developmental Biology, Kobe, Japan; 6 Department of Pharmaceutical Quality Science, Graduate School of Pharmaceutical Sciences, Nagoya City University, Nagoya, Japan; University of Tennessee, United States of America

## Abstract

Human induced pluripotent stem cells (hiPSCs) possess the capabilities of self-renewal and differentiation into multiple cell types, and they are free of the ethical problems associated with human embryonic stem cells (hESCs). These characteristics make hiPSCs a promising choice for future regenerative medicine research. There are significant obstacles, however, preventing the clinical use of hiPSCs. One of the most obvious safety issues is the presence of residual undifferentiated cells that have tumorigenic potential. To locate residual undifferentiated cells, in vivo teratoma formation assays have been performed with immunodeficient animals, which is both costly and time-consuming. Here, we examined three in vitro assay methods to detect undifferentiated cells (designated an in vitro tumorigenicity assay): soft agar colony formation assay, flow cytometry assay and quantitative real-time polymerase chain reaction assay (qRT-PCR). Although the soft agar colony formation assay was unable to detect hiPSCs even in the presence of a ROCK inhibitor that permits survival of dissociated hiPSCs/hESCs, the flow cytometry assay using anti-TRA-1-60 antibody detected 0.1% undifferentiated hiPSCs that were spiked in primary retinal pigment epithelial (RPE) cells. Moreover, qRT-PCR with a specific probe and primers was found to detect a trace amount of Lin28 mRNA, which is equivalent to that present in a mixture of a single hiPSC and 5.0×10^4^ RPE cells. Our findings provide highly sensitive and quantitative in vitro assays essential for facilitating safety profiling of hiPSC-derived products for future regenerative medicine research.

## Introduction

Pluripotent stem cells such as embryonic stem cells and induced pluripotent stem cells have two capabilities: 1) pluripotency: the ability to differentiate into a variety of cells and 2) self-renewal: the ability to undergo numerous cycles of cell division while maintaining their cellular identity. Because of these two characteristics, it has been expected that they would provide new sources for robust and continuous production of a variety of cells and tissues for regenerative medicine/cell therapy. Additionally, hiPSCs offer us a possible solution to the ethical problems and the immune rejection of hESC-derived cells, thus raising novel avenues for patient-specific cell therapy. As previously reported [1], [2], many attempts are currently underway to differentiate hESCs and hiPSCs into various tissues: cardiomyocytes [2], [3], neurons [2], [4], and hepatocytes [5], [6]. It is noteworthy that clinical trials have been conducted with retinal pigment epithelial (RPE) cells derived from hESCs to treat patients with dry age-related macular degeneration and Stargardt's macular dystrophy by Advanced Cell Technology. hiPSCs have also been shown to differentiate into RPE cells, which display functionality both *in vitro* and *in vivo* [7], [8]. Thus, autologous transplant of hiPSC-derived RPE cells holds great promise in the clinical therapy of macular degeneration.

Although hiPSCs overcome immunogenic and ethical barriers, the translation of hiPSCs into the clinical setting faces the same significant problems as those of hESCs. One of the most important issues in the development of a safe pharmaceutical or medical device derived from human pluripotent stem cells is ensuring that the final product does not form tumors after implantation [9]. There are two primary concerns. First, the cell-based product may be unstable and transform to produce a tumor, which is a common problem for any cell-based products, regardless of the cell types of the raw materials. Second, the product derived from human pluripotent stem cells might contain residual undifferentiated stem cells that would eventually proliferate and form a teratoma [10]. Previous reports have shown that several hundred hESCs were sufficient for generating tumors in immunodeficient mice [11], [12]. Hence, to adress the second concern above, it is critical to develop highly sensitive assays for detection of residual undifferentiated stem cells in the final products, and to determine their lower limit of detection (LLOD). An evaluation study of the *in vivo* tumorigenicity assay using severe combined immunodeficiency (SCID) mice has shown that 245 undifferentiated hESCs spiked into 10^6^ feeder fibroblasts produce a teratoma [11]. On the other hand, some *in vitro* assays, such as quantitative real-time polymerase chain reaction (qRT-PCR), flow cytometry and immunohistochemistry, have been used to indicate the undifferentiated state of stem cells with various markers (such as Oct-3/4, Nanog, Sox2, TRA-1-60, TRA-1-81, SSEA-3 and SSEA-4) [13]–[15]. However, it has not been determined how many residual undifferentiated hiPSCs can be detected by these *in vitro* assays.

In this study, to establish a high sensitivity assay for detection of residual undifferentiated hiPSCs in the final product, we evaluated three *in vitro* assays: soft agar colony formation assay, flow cytometry and qRT-PCR. To achieve this goal, these assays were used on cell mixtures that contained defined numbers of undifferentiated hiPSCs in primary RPE cells, and we also tried to determine the LLOD of each assay by using multiple lots of primary RPE cells as backgrounds. Through this process, we revealed that one-step qRT-PCR using probes and primers targeting Lin28 transcripts can detect levels as low as 0.002% residual undifferentiated cells in hiPSC-derived RPE cells.

## Results

### In vitro differentiation of hiPSCs into retinal pigment epithelial cells

**Figure 1:**
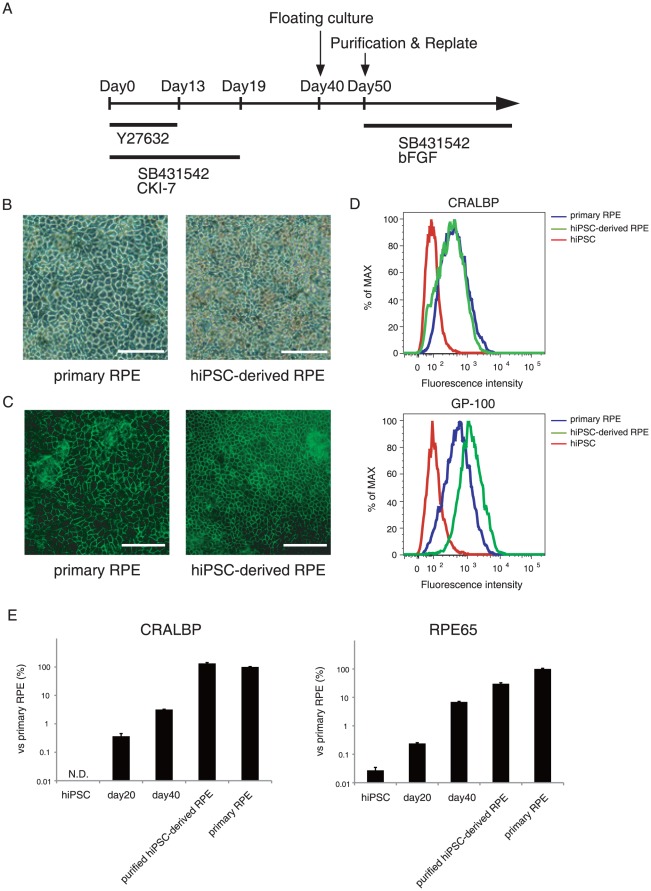
Differentiation of hiPSCs into retinal pigment epithelial cells. (A) Schematic diagram of the culture procedure for retinal differentiation. Photomicrograph (B) and N-cadherin staining (C) shows that both primary RPE cells and hiPSC-derived RPE cells form polygonal, cobblestone-like morphology. Scale bars, 100 µm. (D) Flow cytometry analysis of CRALBP and GP-100 expression in hiPSCs (red), hiPSC-derived RPE cells (green) and primary RPE cells (blue). (E) Time-course analysis of expression of RPE cell markers, CRALBP (left) and RPE65 (right), using qRT-PCR. Error bars represent the standard deviation of the measurements (n = 3).

Minimizing contamination of undifferentiated pluripotent stem cells in cell therapy products is crucial because of the risk of tumorigenesis. To evaluate residual undifferentiated hiPSCs in differentiated cells, it is necessary to determine the LLOD of the hiPSC content in RPE cells. First, we differentiated hiPSCs into RPE cells using the *in vitro* differentiation protocol previously described (Fig. 1A) [7]. The hiPSC-derived RPE cells exhibited polygonal, cobblestone-like morphology, an indication of RPE maturation, which is similar to that of the primary RPE cells (Fig. 1B). Immunocytochemical staining revealed that N-cadherin, the major cadherin expressed in RPE cells [16], showed a distribution to the tight junction of the hiPSC-derived RPE cells, which is consistent with primary RPE cells (Fig. 1C). Moreover, in flow cytometry experiments, a strong expression of the visual cycle protein CRALBP and the melanosomal matrix protein GP-100 was detected in both primary RPE and hiPSC-derived RPE cells compared to in undifferentiated hiPSCs (Fig. 1D). To characterize developmental stages during RPE differentiation, a qRT-PCR assay was used to identify transcript levels of CRALBP and the visual cycle protein RPE65, indicating that CRALBP and RPE65 increased as differentiation progresses and were equally well expressed both in the mature hiPSC-derived RPE cells and primary RPE cells (Fig. 1E). Together, these data showed that mature RPE cells differentiated from hiPSCs possess similar properties to primary RPE cells.

### Soft agar colony formation assay of hiPSCs

**Figure 2:**
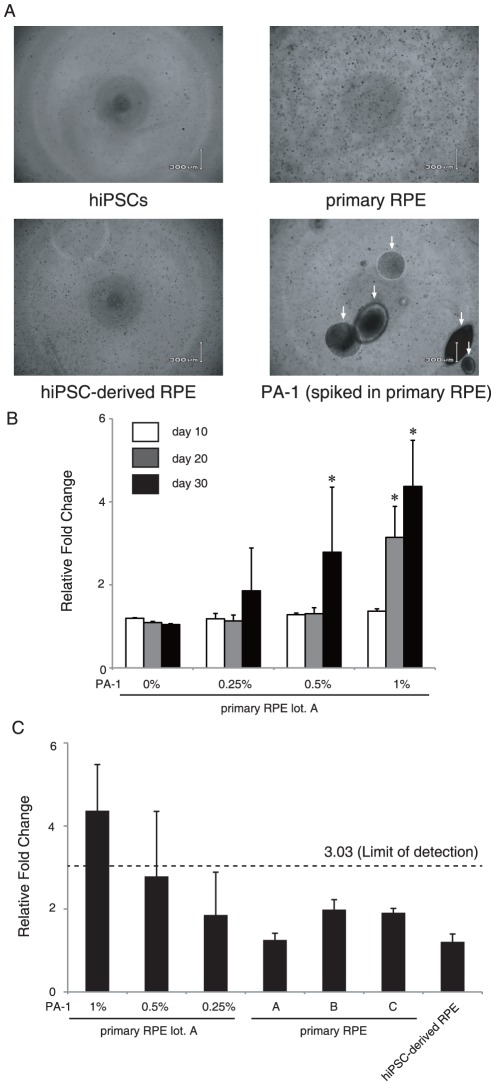
Soft agar colony formation assay of hiPSCs and teratocarcinoma PA-1 cells. (A) Phase-contrast images of hiPSCs, primary RPE cells, hiPSC-derived RPE cells and PA-1cells spiked into primary RPE cells (1%) cultured in soft agar medium for 30 days. Arrows indicate the colonies of PA-1 cells. (B) PA-1 cells (1%, 100 cells; 0.5%, 50 cells; 0.25%, 25 cells; 0%, 0 cells) were spiked into 1.0×10^4^ primary RPE cells and grown in soft agar for 10, 20 and 30 days. Cell growth was quantified using a CytoSelect kit. Results were expressed as a relative fold change of the value of blank well. Statistical significance was determined using two-way ANOVA and Bonferroni's post-hoc test (*P<0.05 compared with the 0% control). (C) HiPSC-derived RPE cells, three lots of primary RPE cells and PA-1 cells spiked into primary RPE cells were grown in soft agar for 30 days. Quantification of the results is described in (B). Limit of detection was calculated as the mean plus 3.3 fold the standard deviation of the measurement of the three lots of primary RPE cells. Error bars represent the standard deviation of the measurements (n = 3).

The soft agar colony formation assay is a general method to monitor anchorage-independent growth, which is considered the most appropriate *in vitro* assay for detecting the malignant transformation of cells [17]. To measure cell transformation quantitatively, we used the CytoSelect 96-well Cell Transformation Assay, as described in the Materials and Methods section. Previous reports have shown that human pluripotent stem cells undergo apoptosis when dissociated into single cells [18]. However, preparation of single cell suspension is quite important because the presence of cell clumps or adjacent cells is critical for growth in an agar medium. Thus, we first sought to clarify whether single hiPSCs grow in the soft agar medium. The soft agar colony formation assay showed that single-hiPSCs could not proliferate in the agar medium. In addition, the ROCK inhibitor Y-27632, which has been reported to inhibit apoptosis [19], did not improve survival of hiPSCs under our conditions (Fig. 2A and S1A). These results demonstrated that the soft agar colony formation assay is not appropriate for detection of undifferentiated hiPSCs in single cell suspension.

Next, we tested whether colony formation of the human ovarian teratocarcinoma cell line PA-1 [20] occurs in an agar medium, since PA-1 cells could mimic possible malignant cells derived from hiPSC. PA-1 cells efficiently formed colonies in soft agar depending on the number of cells (Fig. S1B). On the other hand, primary RPE cells did not grow in a soft agar media with 1.0×10^4^ cells/well, even when cultured for 30 days (Fig. S1C). Based on these results, we aimed to examine the sensitivity of the soft agar colony formation assay to detect PA-1 cells. We spiked 100 (1%), 50 (0.5%), and 25 (0.25%) PA-1 cells into 1×10^4^ primary RPE cells in order to define the minimum number of PA-1 cells required to grow in a soft agar media. The largest number of PA-1 cells (1%) gave rise to detectable colonies within 20 days, whereas lesser numbers of PA-1 cells (0.5% and 0.25%) required 30 days until the colonies become detectable (Fig. 2A and B). We next tried to determine the LLOD of the soft agar colony formation assay. The LLOD of the assay signal was calculated as the mean plus 3.3 fold the standard deviation of the measurement of negative controls [21]. Based on signals from three lots of primary RPE cells as a negative control (1.71±0.40 [fold over the background signal]), the LLOD of the soft agar transformation assay was calculated as 3.03 (Fig. 2C). These results indicate that at least 1% spiked PA-1 cells are necessary for detecting colonies in primary RPE cells using the soft agar transformation assay (4.4±1.1). As shown in Fig. 2C, the hiPSC-derived RPE cells showed no colony formation in the soft agar medium, and the assay signal was lower than the LLOD (1.21±0.19). These results suggest that the contamination of transformed cells, assuming that their anchorage independency is comparable to PA-1, is less than 1% in the hiPSC-derived RPE cells.

### Detection of undifferentiated hiPSCs by flow cytometry

**Figure 3:**
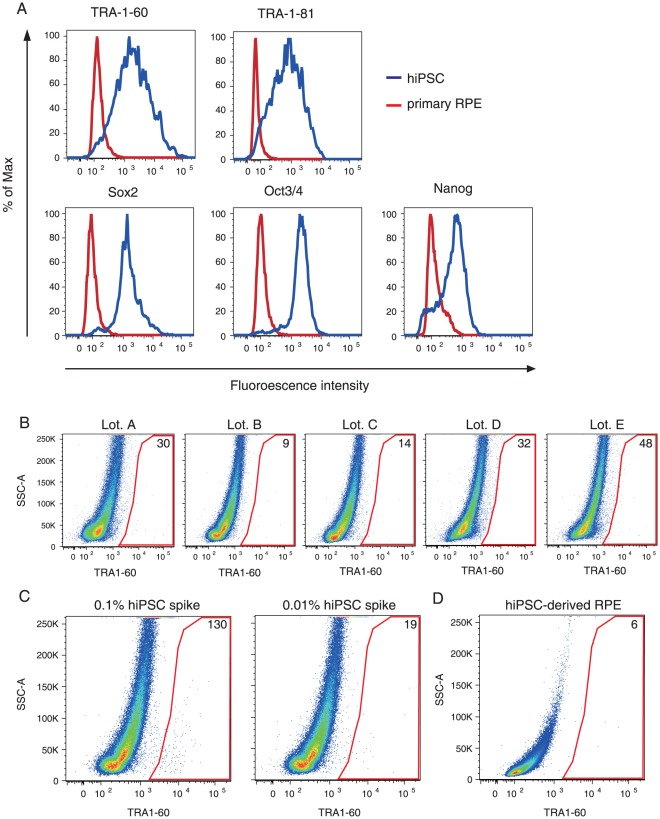
Detection of undifferentiated hiPSCs by flow cytometry assay. (A) Flow cytometry analysis of hiPSCs (blue) and primary RPE cells (red). Cells were fixed, permiabilized and stained with anti-TRA-1-60, anti-TRA-1-81, anti-Sox2, anti-Oct3/4 and anti-Nanog antibodies labeled with fluorophore. (B) Five lots of primary RPE cells were analyzed by flow cytometry with anti-TRA-1-60 antibody. (C) HiPSCs (0.1%, 2.5×10^2^ cells; 0.01%, 25 cells) were spiked into primary RPE cells (2.5×10^5^ cells) and analyzed by flow cytometry with anti-TRA-1-60 antibody. (D) Flow cytometry analysis of hiPSC-derived RPE cells was performed with anti-TRA-1-60 antibody. Ten thousand cells (A) and 1×10^5^ cells (B–D) were used for one assay of flow cytometry analysis. The numbers indicate the quantity of cells contained in the gate.

In the second set of the experiments, we tried to detect residual undifferentiated cells via flow cytometry. Using five antibodies which recognize stem cell marker antigens (Oct3/4, Nanog, Sox2, TRA-1-60, TRA-1-81), we first attempted to identify highly selective markers that distinguish a small population of hiPSCs from primary RPE cells. To minimize nonspecific staining, we used fluorescent conjugated monoclonal antibodies for the flow cytometry. All of the stem cell markers were detected in undifferentiated hiPSCs, but the levels of cell staining differed between the antibodies, presumably attributable to the protein expression in the cells and/or the avidity of the antibodies. The fluorescence histograms of hiPSCs and primary RPE cells showed that anti-Oct3/4, anti-Sox2 and anti-TRA-1-60 antibodies clearly distinguished hiPSCs from RPE cells (Fig. 3A). Because TRA1-60 is not only a marker of undifferentiated hiPSCs but also of embryonal carcinoma [22], we employed anti-TRA-1-60 antibody in further experiments. The TRA-1-60^+^ gate was defined as including at most 0.05% of the primary RPE cells with the highest fluorescence (Fig. 3B). In the flow cytometry, the mean and standard deviation of TRA-1-60^+^ cells in the primary RPE cells (the negative controls) were 26.6 and 15.6 cells/10^5^ cells, respectively, producing an LLOD of 78.1 cells/10^5^ cells (Fig. 3B). To analyze the performance of the system for detection of residual hiPSCs, we spiked 2.5×10^3^ (0.1%) and 2.5×10^2^ (0.01%) hiPSCs into 2.5×10^6^ primary RPE cells and analyzed 1.0×10^5^ cells via flow cytometry using anti-TRA-1-60 antibody. We also confirmed the number of the applied hiPSCs by spiking CFDA-stained hiPSCs (Fig. S2). In the experiment shown in Fig. 3C, 130 and 19 cells were identified as TRA-1-60^+^ cells in 0.1% and 0.01% hiPSCs spiked samples, respectively. These results indicated that at least 0.1% of residual undifferentiated hiPSCs (2.5×10^3^ cells out of 2.5×10^6^ cells) can be detected via the flow cytomerty. Finally, to detect residual undifferentiated cells in hiPSC-derived RPE cells, we tested 1×10^5^ cells and detected six TRA-1-60^+^ cells, suggesting that the population of undifferentiated hiPSCs in the hiPSC-derived RPE cells was no more than 0.1% (Fig. 3D).

### Detection of undifferentiated hiPSCs via qRT-PCR

**Figure 4:**
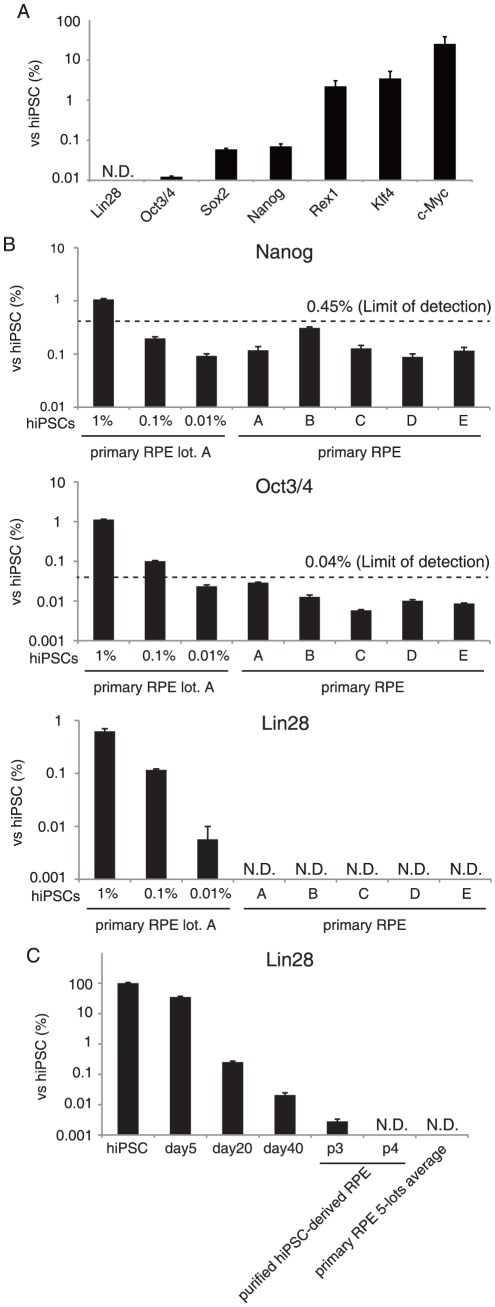
Detection of undifferentiated hiPSCs by qRT-PCR assay. (A) The relative mRNA expressions in primary RPE cells of Lin28, Oct-3/4, Sox2, Nanog, Rex1, Klf4, and c-Myc were determined by qRT-PCR analysis. (B–D) qRT-PCR analysis of hiPSCs spiked into primary RPE cells and five lots of primary RPE cells. Single-cell hiPSCs (1%, 2.5×10^3^ cells; 0.1%, 2.5×10^2^ cells; 0.01%, 25 cells) were spiked into 2.5×10^5^ primary RPE cells, and total RNA was isolated from the mixed cells. The mRNA levels of Nanog (B), Oct3/4 (C) and Lin28 (D) are shown as a relative expression. Limit of detection was calculated as the mean plus 3.3 fold the standard deviation of the measurement of the five lots of primary RPE cells. (E) Lin28 expression of hiPSCs differentiating into RPE and purified hiPSC-derived RPE cells (passage 3 and 4). All values are expressed as mRNA levels relative to those in undifferentiated hiPSCs. Results are means ± standard deviation (n = 3).

We next tested the ability of qRT-PCRs to detect a trace amount of stem cell-specific mRNA. To identify highly selective markers for undifferentiated hiPSCs, we compared the mRNA levels of OCT3/4, KLF4, c-MYC, SOX2, NANOG, LIN28 and REX1 in hiPSCs and primary RPE cells (Fig. 4A). Primary RPE cells were found to endogenously express c-Myc at 25.49% of the levels observed in hiPSC, which was consistent with the previous finding that MEF expressed c-Myc at approximately 20% of levels observed in mouse ES cells [23]. The expression levels of Klf4 and Rex1 in primary RPE cells were 3.51% and 2.23% compared with hiPSCs, respectively. On the other hand, more than a 1000-fold difference between primary RPE cells and hiPSCs was observed in the gene expression of Nanog (0.07%), Sox2 (0.06%), Oct3/4 (0.01%) and Lin28 (not detected). These results suggested that the latter four genes are useful in detecting hiPSCs in RPE cells.

We then conducted two sets of qRT-PCR assays to evaluate the utility of these marker genes to detect spiked hiPSCs in primary RPE cells. We first measured mRNA levels of Nanog, Oct3/4 and Lin28 in the five lots of primary RPE cells (the negative control) to determine the LLOD. The LLODs for Nanog and Oct3/4 mRNAs were 0.45% and 0.04% those of hiPSCs, respectively (Fig. 4B). The LLOD of Lin28 could not be calculated because no fluorescent signal for Lin28 expression was detectable in the primary RPE cells. These results along with experiments spiking 2.5×10^4^ (1%), 2.5×10^3^ (0.1%) and 2.5×10^2^ (0.01%) hiPSCs into 2.5×10^6^ primary RPE cells (Fig. 4B) indicated that measurements of Nanog, Oct3/4 and Lin28 expression by qRT-PCRs could detect at least 1%, 0.1% and 0.01% contamination of residual undifferentiated hiPSCs in RPE cells, respectively.

Finally, we examined whether qRT-PCR for Lin28 was applicable in the detection of residual undifferentiated cells in differentiated RPE cells from hiPSCs. Total RNA (250 ng) extracted from the differentiating cells (day 5, 20, and 40) and purified hiPSC-derived RPE cells (the passage number 3 and 4) were subjected to qRT-PCR analysis. The mRNA level of Lin28 was continuously down-regulated during the differentiation process, being 0.02% that of hiPSCs at day 40. In early passage culture (passage number 3) after purification, Lin28 was still significantly expressed at a level 0.002% that of hiPSCs. From passage 4 onward, however, no detectable level of Lin28 expression was observed in hiPSC-derived RPE cells (Fig. 4C). These results suggest that qRT-PCR analysis for Lin28 detects 0.002% of residual undifferentiated hiPSCs in hiPSC-induced PRE cells, namely, that a single hiPSC in 5.0×10^4^ RPE cells is detectable.

## Discussion

For the clinical use of products derived from human pluripotent stem cells, it is essential to improve both the efficacy and safety of the final product. In order to develop safe hiPSC-based treatments, the hurdle of tumorigenicity arising from undifferentiated cells must be overcome [9]. To address the issue of tumorigenicity, some recent publications have advocated the development of protocols for the derivation of hiPSC [23]–[25] and have outlined methods for the elimination of residual hESCs [26]. However, to date, more than several hundred cells are necessary for human pluripotent stem cells to form a tumor in immunocompromised mice [11]. Therefore, highly sensitive tumorigenicity assays and their standardization are necessary for detecting a small population of residual undifferentiated cells in products derived from human pluripotent stem cells.

In the present study, we evaluated three methods for detection of residual undifferentiated hiPSCs in hiPSCs-derived differentiated cells: soft agar colony formation assay, flow cytometry and qRT-PCR. Table 1 summarizes the advantages and disadvantages of the assays associated with product tumorigenicity. The soft agar colony formation assay is known to be more sensitive in the detection of certain types of tumorigenic cells, compared to *in vivo* methods using immunocompromised mice [27]. However, we found the soft agar colony formation assay unsuitable for detecting residual undifferentiated hiPSCs, presumably attributable to the dissociation-induced apoptosis of hiPSCs [18]. On the other hand, flow cytometry and qRT-PCR assays were found to be able to detect a trace amount of undifferentiated cells. These two assays have been exploited for characterization of stem cell-based products, as well as undifferentiated pluripotent stem cells, but the present study is the first analytical and quantitative approach designed to evaluate the detection of residual undifferentiated cells in products derived from human pluripotent stem cells. The advantage of the flow cytometry assay is that it is able to identify undifferentiated cells. Unfortunately, the results are greatly affected by gating, and only the cells expressing the marker protein are detectable. On the other hand, the advantages of qRT-PCR are its rapidity, quantitativity and high sensitivity, whereas its disadvantage is that only the cells expressing the marker gene are detectable. Although the *in vivo* tumorigenicity assay is costly and time-consuming, it can directly analyze tumor formation in a specific microenvironment where the product is implanted (eg. retina). Therefore, a combination of relevant *in vitro* and *in vivo* assays would be necessary to ensure the safety of a product derived from human pluripotent stem cells. The rationale for the choice of specific assays would be justified, based on their characteristics shown in Table 1.

**Table 1:** Comparison of the tumorigenicity-associated assays.

Assay	Soft agar colony formation assay	Flow cytometry	qRT-PCR	*In vivo* tumorigenicity assay using SCID mice (Reference #11)
Measurement standard	Colony formation	Expression of marker protein for pluripotency	Expression of marker gene for pluripotency	Tumor formation
Purpose	Detection of anchorage independent growth	Detection of undifferentiated pluripotent cells	Detection of undifferentiated pluripotent cells	Detection of tumorigenic or undifferentiated pluripotent cells
Time	30 days	1 day	6 hours	12–16 weeks
Advantage	Inexpensive	Rapid	Rapid and simple	Direct
		Analyzing individual cells	Quantitative	Analyzing tumor formation in a specific microenvironment
			High sensitivity	
Disadvantage	Indirect	Indirect	Indirect	Costly
	Not applicable to hiPSCs	Detecting only the cells that express the known maker molecules	Detecting only the cells that express the known marker genes	Time-consuming
		Gating techniques strongly influence the result		
LLOD	1% of PA-1	0.1% of hiPSC (TRA-1-60)	= <0.002% of hiPSC* (Lin28)	245 undifferentiated hESCs with 10^6^ feeder fibroblasts (0.025%)

* Not based on the calculation found in Reference #21 because the background signal from the negative controls (primary RPE cells) was not detectable.

We have demonstrated that the qRT-PCR assay can successfully detect 0.002% residual undifferentiated hiPSCs in hiPSC-induced RPE cells using Lin28 as a target gene (Fig. 4E). To the best of our knowledge, this qRT-PCR assay, using solely 250 ng of total RNA obtained from approximately 5×10^4^ cells, is the most sensitive of the previously reported methods in detecting undifferentiated pluripotent stem cells. Lin28 is known to specifically inhibit the processing of let-7 miRNAs, which are involved in cell-fate decisions [28]. Interestingly, the aberrant expression of Lin28 transcripts has been recently reported in human germ-cell tumors [29], suggesting that Lin28 is a useful marker of germ-cell malignancy as well as of pluripotency of hiPSCs. Lin28 mRNA gradually decreased in the differentiation process and was completely diminished by passage 4 (Fig. 4C). These observations suggest that Lin28 transcripts are also available for presenting degree of differentiation in hiPSC-derived products because detection of residual Lin28 confirms the contamination of undifferentiated cells even at a late stage of differentiation. Needless to say, the distinct expression of Lin28 could possibly be observed in other normal somatic cells. However, Lin28 is, at least in part, one of the potent markers for detecting incompletely differentiated cells contained in RPE cells derived from pluripotent stem cells.

A great deal of international research is currently being directed at developing regenerative medicine using pluripotent stem cells. Until now, however, little attention has been paid to developing methods to detect undifferentiated cells in pluripotent stem cell-based products. Here, we have revealed that a qRT-PCR method targeting Lin28 can effectively detect a trace amount of hiPSCs in hiPSC-induced RPE cells and shows potential as an *in vitro* tumorigenecity assay of hiPSC-derived cells. We expect our findings to contribute to the process of validation and quality control of hiPSCs-based cell therapy products and to promote the application of regenerative medicine in the treatment of a wide variety of diseases in the near future.

## Materials and Methods

### Cell culture

HiPSC line 201B7 induced by transducing Oct3/4, Sox2, Klf4 and c-Myc [2] was obtained from the RIKEN Cell Bank. Undifferentiated hiPSCs were maintained on mitomycin C-treated SNL cells (a mouse fibroblast STO cell line expressing the neomycin-resistance gene cassette and LIF) in human ES cell culture medium (ReproCell, Japan) supplemented with 4 ng/ml human basic fibroblast growth factor (bFGF; WAKO, Japan). Undifferentiated colonies were passaged as small clumps once in every 5–6 days using CTK solution (ReproCell) and STEMPRO EZPassage (Invitrogen, Carlsbad, CA). Human primary RPE cells were obtained from the Lonza and ScienCell Research Laboratories. The primary RPE cells were maintained in Retinal Pigment Epithelial Cell Basal Medium (Lonza Biologics, Basel, Switzerland) containing supplements (l-glutamine, GA-1000 and bFGF; Lonza). PA-1 cells derived from human ovarian teratocarcinoma (ATCC, Manassas, VA) were maintained on Minimum Essential Medium Eagle medium (Sigma–Aldrich, St. Louis) supplemented with 10% (v/v) fetal bovine serum (FBS; Gibco, Paisley, UK). All cell lines and differentiated cells were grown in a humidified atmosphere of 5% CO_2_ and 95% air at 37°C.

### RPE cell differentiation of hiPSCs

The procedure for differentiating hiPSCs into RPE cells was performed according to the previously described protocol [7] as shown in Figure 1A. The hiPSC clumps were first incubated on poly-d-lysine/gelatin-coated dishes in human ES cell culture medium supplemented with 10 µM Y-27632 (WAKO), 5 µM SB431542 (Sigma–Aldrich) and 3 µM CKI-7 (Sigma–Aldrich) for 1 day. The cells were incubated in a differentiation medium (Glasgow minimum essential medium [GMEM; Invitrogen], 0.1 mM non-essential amino acids, 1 mM sodium pyruvate, and 0.1 mM 2-mercaptoethanol) containing 20% knockout serum replacement (KSR; Invitrogen) for 4 days, then in 15% KSR-containing differentiation medium for 6 days, and finally in 10% KSR-containing differentiation medium for 11–30 days. Y-27632 (10 µM), SB431542 (5 µM) and CKI-7 (3 µM) were added to the differentiation medium for the first 13, 19 and 19 days, respectively. Partially differentiated cells were dissociated with the CTK solution and incubated on non-adhesive dishes (Corning, Corning, NY) in RPE maintenance medium (DMEM:F12 [7∶3] supplemented with B-27 supplement [Invitrogen] and 2 mM l-glutamine [Invitrogen]) for 10 days. The resulting RPE cell aggregates were isolated and replated on CELLstart- (Invitrogen) coated dishes in RPE maintenance medium supplemented with 0.5 µM SB431542 and 10 ng/ml bFGF and we defined this stage as passage 1. The medium was changed every 2–3 days.

### Soft agar colony formation assay

A soft agar colony formation assay was performed using CytoSelect 96-well Cell Transformation Assay kit (Cell Biolabs, San Diego, CA) according to the manufacturer's instructions with slight modification. Prewarmed 25 µl of 2×DMEM medium containing 20% FBS and 25 µl of 1.2% agar solution were mixed and transferred onto a well of 96-well plates, and then incubated at 4°C for 30 min to allow the bottom agar layer to solidify. Single cell suspensions were prepared as described below: 201B7 cells were dissociated with CTK solution to form cell clumps and incubated on gelatin-coated dishes in the presence of 10 µM Y-27632, a ROCK inhibitor, at 37°C for 1 h to separate with feeder cells. After centrifugation, cell pellets were dissociated into single cells with Accutase (Millipore). Primary RPE cells, hiPSC-derived RPE cells, and PA-1 cells were treated with 0.25% trypsin-EDTA solution (Invitrogen) to dissociate. Cells were passed through 40 µm nylon cell strainers (BD Falcon).

Next, 25 µl of single cell suspensions containing the defined number of cells were mixed with 25 µl of 2×DMEM medium containing 20% FBS and 25 µl of 1.2% agar, and placed on the bottom agar layer. The top agar layers were immediately solidified at 4°C for 10 min to avoid false-positive signals derived from gravity-induced adjacent cells in the agar medium. After the addition of 100 µl of DMEM containing 10% FBS to each well, the plates were incubate for 10, 20 and 30 days at 37°C and 5% CO_2_. The medium was changed every 2–3 days. Colonies were lysed and quantified with CyQuant GR dye using a fluorometer equipped with a 485/520 nm filter set (Wallac 1420 ARVOsx mutilabel counter, PerkinElmer, Boston, MA).

### qRT-PCR

Total RNA was isolated from cell cultures using an RNeasy Mini Kit (Qiagen, Hilden, Germany) and treated with DNase I according to the manufacturer's instructions. In the spike study, 201B7 cells and RPE cells were mixed at a defined cell number, before total RNA isolation. qRT-PCR was performed with the QuantiTect Probe one-step RT-PCR Kit (Qiagen) on a 7300 Real-Time PCR System (Applied Biosystems, Foster City, CA). The expression levels of target genes were normalized to those of the GAPDH (glyceraldehyde-3-phosphate dehydrogenase) transcript, which were quantified using TaqMan human GAPDH control reagents (Applied Biosystems). Probes and primers were obtained from Sigma–Aldrich. The sequences of primers and probes used in the present study are listed in Table S1. All qRT-PCR reactions were run at 45 cycles.

### Flow Cytometry

201B7 cells and RPE cells were dissociated into single cells as described above. Cells were fixed with the BD Cytofix fixation buffer (BD Biosciences, Bedford, MA) for 20 min and permeabilized with BD Perm/Wash buffer (BD Biosciences) for 10 min at room temperature. Cells were incubated for 1 h at room temperature with the following primary antibodies and fluorochrome-conjugated antibodies: mouse anti-CRALBP monoclonal 1∶1000 (B2, Thermo Scientific, Roskilde, Denmark); rabbit anti-GP-100 monoclonal 1∶1000 (P14-V, Enzo Life Sciences, Lausen, Switzerland); FITC mouse anti-TRA-1-60 monoclonal 1∶5 (TRA-1-60, BD Pharmingen); PE mouse anti-TRA-1-81 monoclonal 1∶5 (TRA-1-81, BD Pharmingen); PerCP-Cy5.5 mouse anti-Oct3/4 monoclonal 1∶5 (40/Oct-3, BD Pharmingen); Alexa Fluor 647 mouse anti-Sox2 monoclonal 1∶5 (245610, BD Pharmingen); PE mouse anti-Nanog monoclonal 1∶5 (N31–355, BD Pharmingen). Indirect immunostaining was then completed with either donkey-anti-mouse or donkey-anti-rabbit Alexa Fluor 647-conjugated secondary antibodies 1∶1000 (Molecular Probes) for 1 h. Appropriate antibodies were used as a negative control. To obtain fluorescein-labeled hiPSCs, 201B7 cells were incubated with 10 µM carboxyfluorescein diacetate succinimidyl ester (CFDA; Invitrogen) in phosphate buffered saline (PBS) for 8 min, dissociated into a single cell suspension, and then fixed as described above. Stained cells were analyzed with a BD FACSAria II (BD Biosciences). Data retrieved from the sorting was analyzed with Flowjo software 9.3.3 (Tree Star, Ashland, OR).

### Immunocytochemistry

All manipulations were performed at room temperature. Cultured primary and hiPSC-derived RPE cells were fixed with 4% paraformaldehyde in PBS for 20 min at room temperature. After washing with PBS, the cells were permeabilized with 0.2% Triton-X100 in PBS for 15 min and blocked with 2% bovine serum albumin in PBS for 30 min. Samples were incubated for 1 h with mouse anti-N-cadherin monoclonal antibody 1∶1000 (GC-4, Sigma–Aldrich). The cells were washed with PBS and incubated with 1∶1000 Alexa Fluor 488 F(ab′)2 fragment of goat anti-mouse IgG 1∶1000 (Molecular Probes) for 1 h. The samples were mounted with a Vectashield mounting medium containing DAPI (Vector Laboratories, Burlingame, CA) and examined with a Biozero-8000 fluorescence microscope (Keyence, Japan).

## Supporting Information

Figure S1 **Soft agar colony formation assay of hiPSCs, teratocarcinoma PA-1 cells and primary RPE cells.** (A) hiPSCs (10000 cells, 6000cells and 3000 cells/well) were grown in soft agar for 10, 20 and 30 days with 10 µM Y-27632. (B) PA-1 cells (1000, 500, 300, 200, 100, 50, 30 cells/well) were grown in soft agar for 20 days. (C) Primary RPE cells (lot. A, 100,000, 60,000, 30,000 and 10,000 cells/well) were grown in soft agar for 30 days. (A–C) Cell growth was quantified using a CytoSelect kit and the results expressed as a relative fold change of the value of a blank well. Error bars represent the standard deviation of the measurements (n = 3).
(EPS)

Figure S2 **Flow cytometry analysis of spiked hiPSCs cells in primary RPE.** CFDA-stained hiPSCs (1%, 2,500 cells; 0.1%, 250 cells; 0.01%, 25 cells) were spiked into primary RPE (2.5×10^5^ cells) and 1×10^5^ cells were analyzed by flow cytometry. The numbers indicate the quantity of cells contained in the gate.
(EPS)

Table S1 **Probes and primers for qRT-PCR.**
(DOCX)
