# Supplementary material for: Highly Sensitive In Vitro Methods for Detection of Residual Undifferentiated Cells in Retinal Pigment Epithelial Cells Derived from Human iPS Cells
Source: PLoS One. 2012 May 17;7(5):e37342. doi: 10.1371/journal.pone.0037342 (PMC3355139; doi:10.1371/journal.pone.0037342)
Supplement: Table S1 — Probes and primers for qRT-PCR. (DOCX) [file pone.0037342.s003.docx]

**Table S1. Probes and primers for qRT-PCR**

| Gene Probe sequences (5′ →3′) Forward primer sequences (5′→3′) Reverse primer sequences (5′→3′) |
| --- |
| NANOG TGCTGAGGCCTTCTGCGTCACACC CTCAGCTACAAACAGGTGAAGAC TCCCTGGTGGTAGGAAGAGTAAA  OCT3/4 CGGACCACATCCTTCTCGAGCCCAAGC GAAACCCACACTGCAGCAGA TCGCTTGCCCTTCTGGCG  LIN28 CGCATGGGGTTCGGCTTCCTGTCC CACGGTGCGGGCATCTG CCTTCCATGTGCAGCTTACTC  TERT CAGCCTCCAGACGGTGTGCACCAAC CCTGTTTCTGGATTTGCAGGTG GCACACATGCGTGAAACCTG  REX1 AGCAAACACCTGCTGGACTGTGAGCAC CCATCGCTGAGCTGAAACAAA CCTCCAGGCAGTAGTGATCTG  c-MYC TTGTTCCTCCTCAGAGTCGCTGCTGGT GCTCCATGAGGAGACACCG CCACAGAAACAACATCGATTTCTTC  SOX2 CTCGCAGACCTACATGAACGGCTCGC GCGCCCTGCAGTACAACTC CGGACTTGACCACCGAACC  KLF4 ACCTGCGAACCCACACAGGTGAGAAAC CCTACACAAAGAGTTCCCATCTCA CCGTCCCAGTCACAGTGGTA  RPE65 CCACAGAAGGTTCATCCGCACTGATGC CCTCCTGCACAAGTTTGACTTTA TGCCAAATTCTGTTATGACGATCC  CRALBP CGCCACACCTTGCAGAAGGCCAAG CCTGTCTTTGGCCCGTGC GTCTCCTCTCTCTCGTTCAGC |
